# Cyclo­propylammonium 4-iodo­benzoate

**DOI:** 10.1107/S160053681202449X

**Published:** 2012-06-13

**Authors:** Andreas Lemmerer

**Affiliations:** a Centre for Supramolecular Chemistry Research, Department of Chemistry, University of Cape Town, Rondebosch 7701, South Africa

## Abstract

In the title mol­ecular salt, C_3_H_8_N^+^·C_7_H_4_IO_2_^−^, the cyclo­propanaminium cation forms three hydrogen bonds to the 4-iodo­benzoate anion, forming two unique repeating *R*_4_^4^(12) hydrogen-bonding rings that result in one-dimensional hydrogen-bonded columns along the crystallographic *c* axis.

## Related literature

For proton-transfer compounds, see: Kinbara *et al.* (1996 ▶). For hydrogen bonds between primary ammonium cations and a carboxyl­ate anion, see: Lemmerer (2011 ▶). For hydrogen-bond motifs, see: Bernstein *et al.* (1995 ▶).
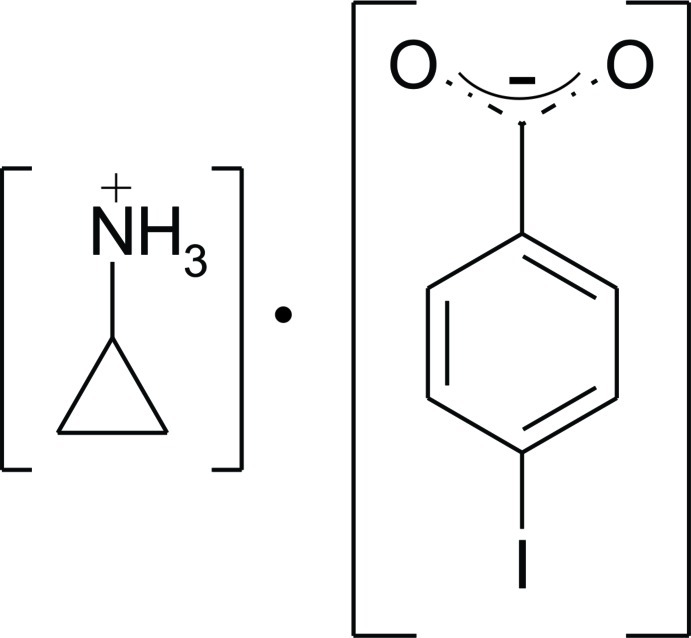


## Experimental

### 

#### Crystal data

C_3_H_8_N^+^·C_7_H_4_IO_2_^−^
*M* *_r_* = 305.11
Orthorhombic, 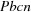

*a* = 30.7877 (6) Å
*b* = 9.7608 (2) Å
*c* = 7.4757 (2) Å
*V* = 2246.54 (9) Å^3^
*Z* = 8
Mo *K*α radiation
μ = 2.83 mm^−1^
*T* = 173 K
0.5 × 0.15 × 0.11 mm

#### Data collection

Bruker APEXII CCD area-detector diffractometer
Absorption correction: integration (*XPREP*; Bruker, 2004 ▶) *T*_min_ = 0.332, *T*_max_ = 0.746
11693 measured reflections
2705 independent reflections
2103 reflections with *I* > 2σ(*I*)
*R*_int_ = 0.037

#### Refinement

*R*[*F* ^2^ > 2σ(*F* ^2^)] = 0.023
*wR*(*F* ^2^) = 0.047
*S* = 0.97
2705 reflections
139 parameters
H atoms treated by a mixture of independent and constrained refinement
Δρ_max_ = 0.42 e Å^−3^
Δρ_min_ = −0.79 e Å^−3^

### 

Data collection: *APEX2* (Bruker, 2005 ▶); cell refinement: *SAINT-Plus* (Bruker, 2004 ▶); data reduction: *SAINT-Plus* and *XPREP* (Bruker 2004 ▶); program(s) used to solve structure: *SHELXS97* (Sheldrick, 2008 ▶); program(s) used to refine structure: *SHELXL97* (Sheldrick, 2008 ▶); molecular graphics: *ORTEP-3 for Windows* (Farrugia, 1997 ▶) and *DIAMOND* (Brandenburg, 1999 ▶); software used to prepare material for publication: *WinGX* (Farrugia, 1999 ▶) and *PLATON* (Spek, 2009 ▶).

## Supplementary Material

Crystal structure: contains datablock(s) global, I. DOI: 10.1107/S160053681202449X/bt5937sup1.cif

Structure factors: contains datablock(s) I. DOI: 10.1107/S160053681202449X/bt5937Isup2.hkl

Supplementary material file. DOI: 10.1107/S160053681202449X/bt5937Isup3.mol

Supplementary material file. DOI: 10.1107/S160053681202449X/bt5937Isup4.cml

Additional supplementary materials:  crystallographic information; 3D view; checkCIF report

## Figures and Tables

**Table 1:** Hydrogen-bond geometry (Å, °)

*D*—H⋯*A*	*D*—H	H⋯*A*	*D*⋯*A*	*D*—H⋯*A*
N1—H1*A*⋯O1	0.86 (3)	1.95 (3)	2.807 (3)	173 (2)
N1—H1*B*⋯O1^i^	0.95 (2)	1.90 (2)	2.807 (2)	161 (2)
N1—H1*C*⋯O2^ii^	0.83 (3)	1.92 (3)	2.739 (2)	171 (2)

Symmetry codes: (i) 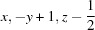; (ii) 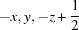.

**Fig. 1.**
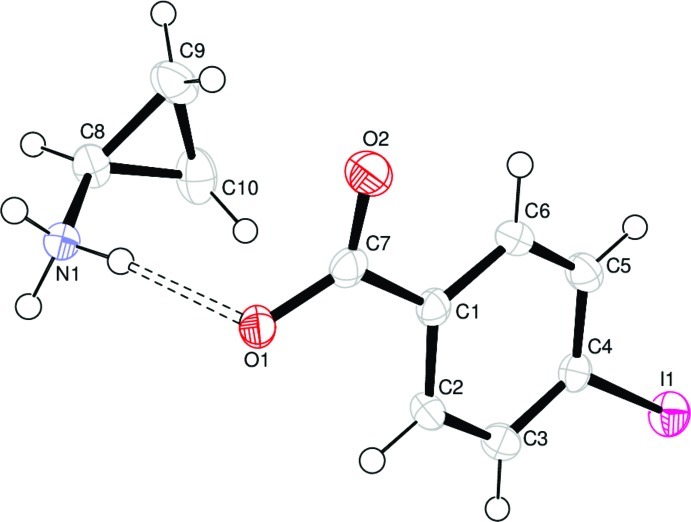
The asymmetric unit and atom numbering scheme of the title compound. Displacement ellipsoids are shown at the 50% probability level and H atoms are shown as small spheres of arbitrary radii.

**Fig. 2.**
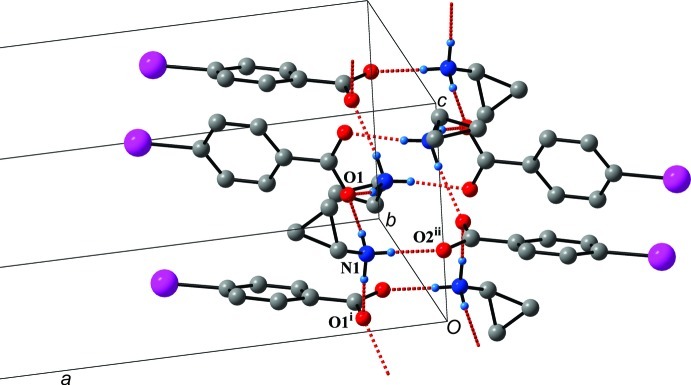
The hydrogen bonding pattern of the title compound. H atoms not involved in hydrogen bonding are omitted for clarity. Atoms marked with superscript i and ii are at the symmetry positions (-x, -y + 1, z - 1/2) and (-x, y, -z + 1/2) respectively.

## supplementary crystallographic information

### Comment

Ammonium carboxylate salts are molecular salts formed by mixing a primary amine and a carboxylic acid containing molecule, thus resulting in proton transfer from the acid to the amine (Kinbara *et al.*, 1996). This forms a primary ammonium cation and a carboxylate anion. The three H atoms on the cation can then form three charge-assisted hydrogen bonds to the two O atoms on the anion. In the literature, three kinds of hydrogen bonded rings are most commonly formed by these hydrogen bonds, described using graph-set notation (Bernstein *et al.*, 1995): *R*^2^_4_(8), *R*^3^_4_(10) and *R*^4^_4_(12) (Lemmerer, 2011).

In molecular salt (I), shown in Fig. 1, formed by dissolving cyclopropylamine and *p*-iodobenzoic acid in methanol, only a *R*^4^_4_(12) ring is formed. However, two such rings are formed, one by using the N1—H1A···O1 and N1—H1B···O1 hydrogen bonds, and the second one by using the N1—H1B···O1 and N1—H1C···O2 hydrogen bonds. As the N1—H1B···O1 hydrogen bond is common to both rings, a repetition of the two types of rings results, forming a 1-D hydrogen bonded column along the *c* axis (Fig. 2).

### Experimental

All chemicals were purchased from commercial sources and used as received. (I) was prepared by slowly evaporating a solution of cyclopropylamine (0.050 g, 0.88 mmol) and *p*-iodobenzoic acid (0.217 g, 0.886 mmol) dissolved in 5 ml methanol.

### Refinement

All C—H atoms were refined using a riding model, with a distance of 0.95 Å (Ar—H), 0.99 Å, (CH_2_) and 1.00 Å, (CH) and *U*_iso_(H) = 1.2*U*_eq_(C). N—H atoms on the ammonium group were located in the difference Fourier map and their coordinates and isotropic displacement parameters were refined freely.

### Crystal data

**Table:**

C_3_H_8_N^+^·C_7_H_4_IO_2_^−^	*F*(000) = 1184
*M**_r_* = 305.11	*D*_x_ = 1.804 Mg m^−^^3^
Orthorhombic, *P**b**c**n*	Mo *K*α radiation, λ = 0.71073 Å
Hall symbol: -P 2n 2ab	Cell parameters from 4806 reflections
*a* = 30.7877 (6) Å	θ = 2.7–28.1°
*b* = 9.7608 (2) Å	µ = 2.83 mm^−^^1^
*c* = 7.4757 (2) Å	*T* = 173 K
*V* = 2246.54 (9) Å^3^	Needle, colourless
*Z* = 8	0.5 × 0.15 × 0.11 mm

### Data collection

**Table:**

Bruker APEXII CCD area-detector diffractometer	2103 reflections with *I* > 2σ(*I*)
ω scans	*R*_int_ = 0.037
Absorption correction: integration (*XPREP*; Bruker, 2004)	θ_max_ = 28°, θ_min_ = 2.2°
*T*_min_ = 0.332, *T*_max_ = 0.746	*h* = −37→40
11693 measured reflections	*k* = −12→11
2705 independent reflections	*l* = −9→9

### Refinement

**Table:**

Refinement on *F*^2^	0 restraints
Least-squares matrix: full	H atoms treated by a mixture of independent and constrained refinement
*R*[*F*^2^ > 2σ(*F*^2^)] = 0.023	*w* = 1/[σ^2^(*F*_o_^2^) + (0.0206*P*)^2^] where *P* = (*F*_o_^2^ + 2*F*_c_^2^)/3
*wR*(*F*^2^) = 0.047	(Δ/σ)_max_ = 0.001
*S* = 0.97	Δρ_max_ = 0.42 e Å^−^^3^
2705 reflections	Δρ_min_ = −0.79 e Å^−^^3^
139 parameters	

### Special details

**Table:**

Experimental. Numerical integration absorption corrections based on indexed crystal faces were applied using the *XPREP* routine (Bruker, 2004)
Geometry. All e.s.d.'s (except the e.s.d. in the dihedral angle between two l.s. planes) are estimated using the full covariance matrix. The cell e.s.d.'s are taken into account individually in the estimation of e.s.d.'s in distances, angles and torsion angles; correlations between e.s.d.'s in cell parameters are only used when they are defined by crystal symmetry. An approximate (isotropic) treatment of cell e.s.d.'s is used for estimating e.s.d.'s involving l.s. planes.

### Fractional atomic coordinates and isotropic or equivalent isotropic displacement parameters (Å^2^)

**Table:**

	*x*	*y*	*z*	*U*_iso_*/*U*_eq_	
C1	0.12089 (7)	0.65046 (19)	0.5124 (3)	0.0213 (4)	
C2	0.14737 (7)	0.5436 (2)	0.4583 (3)	0.0247 (5)	
H2	0.1355	0.4708	0.3895	0.03*	
C3	0.19096 (7)	0.5418 (2)	0.5035 (3)	0.0268 (5)	
H3	0.2089	0.4674	0.4683	0.032*	
C4	0.20809 (7)	0.6497 (2)	0.6003 (3)	0.0225 (4)	
C5	0.18231 (7)	0.7577 (2)	0.6552 (3)	0.0262 (5)	
H5	0.1944	0.8312	0.7221	0.031*	
C6	0.13877 (7)	0.7572 (2)	0.6114 (3)	0.0241 (5)	
H6	0.1208	0.8306	0.6493	0.029*	
C7	0.07329 (7)	0.6516 (2)	0.4662 (3)	0.0235 (5)	
O1	0.06027 (5)	0.56982 (14)	0.34560 (18)	0.0263 (3)	
O2	0.04919 (5)	0.73454 (15)	0.5467 (2)	0.0332 (4)	
I1	0.274626 (5)	0.652799 (16)	0.66287 (2)	0.03283 (6)	
C8	0.05737 (7)	0.8095 (2)	−0.0226 (3)	0.0257 (5)	
H8	0.0536	0.8472	−0.146	0.031*	
C9	0.05800 (8)	0.9120 (2)	0.1242 (3)	0.0353 (6)	
H9A	0.0452	0.8852	0.2405	0.042*	
H9B	0.0542	1.0096	0.0918	0.042*	
C10	0.09938 (8)	0.8420 (2)	0.0668 (3)	0.0383 (6)	
H10A	0.1209	0.8967	−0.0008	0.046*	
H10B	0.1119	0.7723	0.1479	0.046*	
N1	0.03677 (6)	0.67743 (18)	0.0111 (3)	0.0227 (4)	
H1A	0.0424 (9)	0.650 (2)	0.118 (4)	0.042 (8)*	
H1B	0.0454 (7)	0.607 (2)	−0.068 (3)	0.034 (7)*	
H1C	0.0101 (8)	0.686 (2)	−0.001 (3)	0.032 (7)*	

### Atomic displacement parameters (Å^2^)

**Table:**

	*U*^11^	*U*^22^	*U*^33^	*U*^12^	*U*^13^	*U*^23^
C1	0.0246 (10)	0.0218 (10)	0.0174 (10)	−0.0011 (9)	−0.0012 (9)	0.0029 (9)
C2	0.0308 (12)	0.0221 (11)	0.0214 (11)	−0.0006 (9)	−0.0023 (9)	−0.0045 (9)
C3	0.0282 (11)	0.0266 (11)	0.0255 (11)	0.0046 (9)	0.0017 (10)	−0.0037 (10)
C4	0.0212 (10)	0.0273 (11)	0.0190 (10)	−0.0010 (9)	−0.0001 (9)	0.0021 (10)
C5	0.0287 (11)	0.0240 (11)	0.0258 (12)	−0.0040 (9)	−0.0015 (10)	−0.0028 (10)
C6	0.0265 (11)	0.0207 (11)	0.0249 (11)	0.0018 (9)	−0.0003 (9)	−0.0036 (9)
C7	0.0287 (11)	0.0199 (10)	0.0220 (11)	−0.0018 (9)	−0.0040 (9)	0.0065 (10)
O1	0.0329 (8)	0.0222 (8)	0.0238 (8)	−0.0030 (6)	−0.0087 (7)	0.0008 (7)
O2	0.0240 (8)	0.0341 (9)	0.0416 (10)	0.0048 (7)	−0.0064 (7)	−0.0119 (8)
I1	0.02229 (8)	0.04054 (10)	0.03566 (10)	−0.00054 (6)	−0.00137 (7)	−0.00002 (8)
C8	0.0310 (12)	0.0249 (11)	0.0212 (11)	−0.0076 (9)	0.0002 (10)	0.0037 (9)
C9	0.0491 (16)	0.0231 (12)	0.0337 (14)	−0.0057 (11)	0.0002 (12)	−0.0024 (11)
C10	0.0373 (14)	0.0449 (15)	0.0327 (14)	−0.0172 (12)	−0.0037 (11)	0.0041 (12)
N1	0.0240 (10)	0.0213 (10)	0.0229 (11)	0.0007 (8)	−0.0038 (9)	−0.0004 (8)

### Geometric parameters (Å, º)

**Table:**

C1—C2	1.384 (3)	C7—O1	1.269 (2)
C1—C6	1.391 (3)	C8—N1	1.459 (3)
C1—C7	1.506 (3)	C8—C9	1.485 (3)
C2—C3	1.384 (3)	C8—C10	1.490 (3)
C2—H2	0.95	C8—H8	1
C3—C4	1.382 (3)	C9—C10	1.508 (3)
C3—H3	0.95	C9—H9A	0.99
C4—C5	1.383 (3)	C9—H9B	0.99
C4—I1	2.101 (2)	C10—H10A	0.99
C5—C6	1.380 (3)	C10—H10B	0.99
C5—H5	0.95	N1—H1A	0.86 (3)
C6—H6	0.95	N1—H1B	0.95 (2)
C7—O2	1.253 (2)	N1—H1C	0.83 (3)
			
C2—C1—C6	119.13 (19)	C9—C8—C10	60.91 (15)
C2—C1—C7	120.76 (18)	N1—C8—H8	115.8
C6—C1—C7	120.11 (18)	C9—C8—H8	115.8
C3—C2—C1	120.59 (19)	C10—C8—H8	115.8
C3—C2—H2	119.7	C8—C9—C10	59.71 (14)
C1—C2—H2	119.7	C8—C9—H9A	117.8
C4—C3—C2	119.3 (2)	C10—C9—H9A	117.8
C4—C3—H3	120.4	C8—C9—H9B	117.8
C2—C3—H3	120.4	C10—C9—H9B	117.8
C3—C4—C5	121.2 (2)	H9A—C9—H9B	114.9
C3—C4—I1	119.99 (15)	C8—C10—C9	59.38 (15)
C5—C4—I1	118.84 (15)	C8—C10—H10A	117.8
C6—C5—C4	118.9 (2)	C9—C10—H10A	117.8
C6—C5—H5	120.5	C8—C10—H10B	117.8
C4—C5—H5	120.5	C9—C10—H10B	117.8
C5—C6—C1	120.91 (19)	H10A—C10—H10B	115
C5—C6—H6	119.5	C8—N1—H1A	110.5 (16)
C1—C6—H6	119.5	C8—N1—H1B	114.5 (14)
O2—C7—O1	124.1 (2)	H1A—N1—H1B	107 (2)
O2—C7—C1	118.10 (19)	C8—N1—H1C	108.7 (16)
O1—C7—C1	117.78 (19)	H1A—N1—H1C	109 (2)
N1—C8—C9	118.27 (19)	H1B—N1—H1C	107 (2)
N1—C8—C10	119.23 (19)		
			
C6—C1—C2—C3	0.7 (3)	C2—C1—C6—C5	0.2 (3)
C7—C1—C2—C3	−178.98 (19)	C7—C1—C6—C5	179.90 (19)
C1—C2—C3—C4	−1.4 (3)	C2—C1—C7—O2	166.03 (19)
C2—C3—C4—C5	1.1 (3)	C6—C1—C7—O2	−13.6 (3)
C2—C3—C4—I1	−177.88 (16)	C2—C1—C7—O1	−15.2 (3)
C3—C4—C5—C6	−0.2 (3)	C6—C1—C7—O1	165.19 (18)
I1—C4—C5—C6	178.79 (15)	N1—C8—C9—C10	109.6 (2)
C4—C5—C6—C1	−0.5 (3)	N1—C8—C10—C9	−108.0 (2)

### Hydrogen-bond geometry (Å, º)

**Table:**

*D*—H···*A*	*D*—H	H···*A*	*D*···*A*	*D*—H···*A*
N1—H1*A*···O1	0.86 (3)	1.95 (3)	2.807 (3)	173 (2)
N1—H1*B*···O1^i^	0.95 (2)	1.90 (2)	2.807 (2)	161 (2)
N1—H1*C*···O2^ii^	0.83 (3)	1.92 (3)	2.739 (2)	171 (2)

Symmetry codes: (i) *x*, −*y*+1, *z*−1/2; (ii) −*x*, *y*, −*z*+1/2.
